# Cost-effective screening strategy to prevent venous thromboembolism in combined oral contraceptive users

**DOI:** 10.3389/fendo.2025.1559162

**Published:** 2025-06-12

**Authors:** Jonathan Douxfils

**Affiliations:** ^1^ Research Unit in Clinical Pharmacology and Toxicology (URPC), NAmur Research Institute for LIfe Sciences (NARILIS), Faculty of Medicine, University of Namur, Namur, Belgium; ^2^ QUALIresearch, Qualiblood s.a, Liège, Belgium; ^3^ Department of Biological Hematology, Centre Hospitalier Universitaire Clermont-Ferrand, Hôpital Estaing, Clermont-Ferrand, France

**Keywords:** combined oral contraceptives, venous thromboembolism, thrombophilia screening, cost-effectiveness, normalized activated protein c sensitivity ratio

## Abstract

Venous thromboembolism (VTE), encompassing deep vein thrombosis (DVT) and pulmonary embolism (PE), is a leading cause of global morbidity and mortality, with a significant societal and economic burden. Combined oral contraceptives (COCs) increase VTE risk by 2- to 6-fold, resulting in approximately 22,925 cases annually in the European Economic Area (EEA). Despite the high associated healthcare costs, which may reach 2.5 billion EUR annually, current international guidelines, including those from the World Health Organization (WHO) and the Faculty of Sexual and Reproductive Healthcare (FSRH), discourage routine thrombophilia screening prior to COC prescription, citing low cost-effectiveness, low prevalence of thrombophilia, and potential unintended consequences, such as reduced contraceptive use. Recent advancements in screening technology challenge these guidelines. The normalized Activated Protein C sensitivity ratio (nAPCsr) assay, a low-cost tool capable of detecting both inherited thrombophilia and acquired COC-induced activated protein C (APC) resistance, offers a promising strategy for targeted screening. Economic models estimate that implementing nAPCsr-based screening could prevent up to 13,500 VTE cases annually, leading to 1.5 billion EUR in annual healthcare savings. Additionally, nAPCsr-guided contraceptive counseling enables personalized decision-making, directing high-risk women toward safer contraceptive options, such as progestin-only pills or COCs containing natural estrogens (estradiol or estetrol), which present a lower thrombotic risk. This manuscript emphasizes the necessity of updating current prevention strategies by integrating innovative screening tools like the nAPCsr assay. By addressing both direct healthcare costs and indirect costs related to productivity loss and long-term complications, such a strategy could improve patient safety, reduce the financial burden on healthcare systems, and promote equitable access to safer contraceptive methods. Furthermore, targeted screening could alleviate the underrepresentation of high-risk women in current cost estimates and significantly mitigate the societal impact of COC-associated VTE. In light of these findings, reconsidering current policy recommendations appears essential to facilitate evidence-based, cost-effective prevention of COC-related thrombotic events, ultimately enhancing public health outcomes.

## Introduction

Venous thromboembolism (VTE), encompassing deep vein thrombosis (DVT) and pulmonary embolism (PE), is a leading cause of global morbidity, mortality, and healthcare burden (1). With an annual incidence rate ranging from 2 to 30 cases per 10,000 individuals in Western populations, VTE stands as the third most frequent cardiovascular disease, following ischemic heart disease and stroke (2, 3). The condition carries immediate life-threatening risks, including PE, and predisposes survivors to chronic complications such as post-thrombotic syndrome (PTS) and chronic thromboembolic pulmonary hypertension, significantly degrading health related quality of life (HRQoL) and increasing healthcare costs (4, 5).

Venous thromboembolism imposes an extensive societal burden through both direct and indirect costs. In Europe, the direct annual VTE-related healthcare expenditures range between 1.5 billion EUR and 13.2 billion EUR depending on the model used to estimate costs (4), with hospitalizations accounting for the majority of direct medical expenses (2, 4). Nevertheless, the models developed in this study did not include loss of productivity and other indirect costs that could be attributed to the disease, significantly increasing the real cost burden of VTE (4). So, recurrent VTE, which affects 30% of patients within 10 years, further compounds the economic and human burden (5, 6). Survivors of PE often experience lasting impairments in physical performance and HRQoL, underscoring the need for improved preventive measures (7, 8).

Combined oral contraceptives (COCs) are a significant, modifiable contributor to the VTE burden, increasing the risk of VTE by 2- to 6-fold (9, 10). This risk is less observed with progestin-only pills (POP) (11). However, estrogens play a crucial role in contraception by suppressing ovulation, stabilizing the endometrium, and enhancing the contraceptive efficacy of progestins (12). Their negative feedback on the hypothalamic-pituitary axis inhibits gonadotropin secretion, thereby reducing follicular maturation and ovulation, which significantly increases the effectiveness of COCs (12). Moreover, estrogen contributes to cycle control by preventing irregular shedding of the endometrium, thus minimizing unscheduled bleeding, a frequent cause of contraceptive discontinuation (12). While ethinyl estradiol (EE) has historically been the predominant estrogen in COCs, concerns over its dose-dependent impact on coagulation factors and associated thrombotic risks have driven the search for safer alternatives. Newer estrogens, such as estradiol (E2), estradiol valerate (E2V), and estetrol (E4), exhibit reduced hepatic impact and improved tolerability, with E4 demonstrating promising cycle control and a neutral effect on thrombin generation (13, 14). Estrogens also play a crucial role in bone mass acquisition during adolescence and young adulthood, a period critical for achieving peak bone mineral density (BMD). Estrogen exerts its effects by inhibiting bone resorption and promoting bone formation, thereby contributing to optimal skeletal development and long-term bone health (15). Studies indicate that estrogen deficiency during adolescence, whether due to medical conditions, lifestyle factors, or use of estrogen-free contraceptive methods, may impair bone accrual and increase the risk of osteoporosis later in life (14, 15). Therefore, the inclusion of estrogens in hormonal contraceptives is essential when no contraindication exists wince it offers multiple health benefits but the management of the thrombotic risk should be reappraised according to the latest evidence.

Indeed, with over 150 million women globally using COCs (16), the associated risks translate into substantial morbidity. Each year, an estimated 22,925 VTE cases in the European Economic Area (EEA) are attributed to COC use, highlighting a profound societal impact (17, 18). With an average annual incidence of VTE around 5–16 cases per 10,000 women-year in ethinylestradiol (EE)-containing COC, the economic consequences of managing COC-associated VTE are staggering (19). However, most cost-evaluation studies did not include the indirect cost related to e.g. the loss of productivity in this young population (2, 4, 5, 20). Nevertheless, and interestingly, a Norwegian general working-age population study revealed that the crude incidence rate of work-related disability after VTE was 37.5 (95%CI: 29.7–47.3) per 1,000 person-years, versus 13.5 (13.2–13.7) per 1,000 person-years among those without VTE (21). In the same study, subjects with unprovoked VTE had a 52% higher risk of work-related disability than those without VTE (HR 1.52, 95%CI 1.09–2.14) (21). Considering the younger age of patients with COC-associated VTE, i.e. a median age of 33 years old in the START registry for women with COC-associated VTE (22) versus 45 years old in this Norwegian general working-age population study (21), indirect cost may be underrepresented in current cost estimation models.

In a Danish study of 74,137 participants aged 18 to 90+, the three-year attributable societal VTE-event costs have been estimated to be 42,780 EUR with 53% of these costs appearing in the first year following the VTE. Costs estimation for major bleedings resulting from secondary thromboprophylaxis were 51,168 EUR with 46% of these costs appearing in the first year following the VTE (6). Based on these cost estimates and considering a 19.7% price increase due to the inflation during the 2015–2024 period, the annual societal financial burden of COC-associated VTE may reach up to approximately 2.578 billion EUR in the European Economic Area (EEA) (6, 17). It is also important to consider that the 18–50 years category represented 24.7% of this Danish study, leading to a potential underestimation of the loss of productivity cost and the long-term burden related to the VTE event. Knowing that production loss represents the largest percentage of VTE costs, i.e. 47% of the total cost in the first year after diagnosis, followed by 73% in the second year after diagnosis and 78% in the third year after diagnosis in the Danish study (6), the true annual societal financial burden of COC-associated VTE may even be higher in this young population.

These figures illuminate the pressing need for targeted prevention strategies, particularly as COCs are often prescribed to young, otherwise healthy women to prevent pregnancy, a non-life-threatening condition. The elevated risk of VTE, even when minimized by using the safest COC association according to the FSRH, i.e. EE in association with levonorgestrel (23), remains unacceptably high in this population with an annual incidence of VTE estimated between 5 to 16 per 10,000 women-year (3, 19). This risk is furthermore increased in women with concomitant coagulopathies with odds ratio for VTE risk compared to non-users comprised between 7.4 to 44.4-fold (24). Chronic complications such as post-thrombotic syndrome (PTS), recurrent VTE, and other sequelae significantly exacerbate these costs, necessitating a holistic and preventive approach (2, 4). For example, the risk of recurrence after a first VTE event associated with the use of COC has been estimated around 120 to 160 VTE per 10,000 patient-years after stopping both anticoagulation and hormonal contraceptive use (25, 26), representing a 50 to 65-fold increase compared to non-user of contraceptive (3, 19).

Importantly and as highlighted in the Survey on Anticoagulated Patients Register (START) registry, an Italian multicenter observational registry designed to collect data on patients receiving anticoagulant therapy (27), 60.7% of COC-associated VTE cases occur in women with predisposing prothrombotic conditions, such as Factor V Leiden (FVL) mutation, prothrombin gene mutation, antiphospholipid syndrome or natural anticoagulant deficiency (22). A family history of VTE played a role in only less than 15% of these COC-associated VTE cases, underlying the need of different prevention strategies than those currently recommended by international guidelines for COC prescription (22, 23, 28–30). These observations underscore the importance of integrating both thrombophilia screening and familial risk assessments into contraceptive counseling. Moreover, individualized risk stratification, guided by updated screening methodologies, such as the normalized Activated Protein C sensitivity ratio (nAPCsr – more information in **Table 1**) (31) and the replacement of synthetic estrogens like EE by natural estrogens like estradiol (E2) and estetrol (E4), offers a promising avenue for reducing COC-associated VTE incidence and its associated burdens (17, 18, 22, 32, 33). The nAPCsr assay can be qualified as a powerful tool for quantifying resistance to activated protein C (APC), a critical endogenous anticoagulant mechanism. Elevated nAPCsr values are indicative of heightened thrombotic susceptibility, particularly in women carrying thrombophilia mutations (31). Beyond its utility in detecting inherited thrombophilia, the nAPCsr assay also identifies acquired APC resistance associated with COC use, making it uniquely suited to stratify risk in the context of hormonal contraception (17, 34).

**Table 1:** Summary of key characteristics of the normalized Activated Protein C sensitivity ratio (nAPCsr).

How is the nAPCsr measured? (31) The nAPCsr test is based on a TGA using the ETP. It involves: Measuring ETP in PPP, both in the presence and absence of APC. Normalizing the ETP ratio obtained from the patient’s sample using a reference plasma composed of healthy men and women not on COCs, without thrombophilia or coagulation abnormalities, and not taking coagulation-interfering drugs. Practical application of the test (31, 34, 41, 49, 60) Sample type: - The test requires a blood sample collected in sodium citrate tubes, processed to obtain PPP. Turnaround time: - The assay can be performed within 1 hour, provided the laboratory is equipped with a CAT or an automated ST-Genesia system. Interpretation of results: - The test helps to detect pre-existing coagulopathies before prescribing COC. - The test does not provide a precise individual risk percentage for VTE but can be used to classify women as normo-responsive or hyper-responsive to COCs based on COC association related threshold (**Figure 2**). - The test can however provide an estimate of the VTE risk for a particular COC at the population level. Comparison with traditional thrombophilia screening (34) Activated partial thromboplastin time-based APC resistance: - Historically used for FVL diagnosis, with high sensitivity in homozygous carriers. - Less effective for acquired APC resistance, such as that induced by hormonal contraception. - Less suitable for detecting mild or transient hypercoagulable states. Normalized APC sensitivity ratio: - More precise for acquired APC resistance, particularly in the context of COC-induced thrombophilia. - Allows quantitative assessment, making it possible to compare different contraceptive formulations. - Can be integrated into population risk assessment models, enabling better stratification of thrombotic risk. - Provides the initial thrombin generation data which can be used to detect additional coagulation abnormalities. Clinical utility and limitations of nAPCsr Strengths: - Reliable for population-based risk assessment: nAPCsr can effectively estimate the thrombotic potential of different COCs. - Can be used for patient stratification: Identifies women who are normo-responsive or hyper-responsive to COCs, helping guide contraceptive choices. - Detects underlying coagulation abnormalities: May be useful in screening for coagulopathies before initiating COCs. Limitations: - Not a precise individual risk predictor: VTE risk is multifactorial, and nAPCsr does not account for all factors contributing the risk of VTE like BMI, lifestyle, or other non-coagulation-related risk factors. - Limited routine availability: While the ST-Genesia platform improves accessibility, the test still requires dedicated equipment, which may not yet be available in all laboratories.

APC, activated protein C; aPTT, activated Partial Thromboplastin Time; BMI, body mass index; CAT, calibrated automated thrombogram; COC, combined oral contraceptive; ETP, endogenous thrombin potential; FVL, factor V Leiden; nAPCsr, normalized Activated Protein C sensitivity ratio; PPP, platelet poor plasma; TGA, thrombin generation assay; VTE, venous thromboembolism.

This opinion paper attempts to examine how a new screening functional coagulation strategy can minimize the multifaceted burden of COC-associated VTE from medical and economic perspectives, emphasizing the interplay between direct costs (e.g., hospitalization, acute treatments, and follow-up care) and indirect costs (e.g., loss of productivity and long-term disability). By contextualizing COC-associated VTE as a preventable yet economically significant condition, this manuscript aim to highlight the necessity of efficient and innovative prevention strategies and resource allocation to alleviate the profound societal and healthcare impacts of COC-associated VTE.

## Why not screening for thrombophilia before prescribing the pill

The current guidelines from leading organizations such as the World Health Organization (WHO), the Centers for Disease Control and Prevention (CDC), the American Society of Hematology (ASH), and the Faculty of Sexual and Reproductive Healthcare (FSRH) recommend against routine thrombophilia screening before prescribing COCs (23, 28–30). These guidelines cite low cost-effectiveness, practicality, low prevalence of thrombophilia conditions, and potential unintended consequences as the primary reasons for this position. However, these recommendations may warrant reevaluation considering emerging evidence and technological advancements.

One of the major arguments against routine thrombophilia screening is the high cost of traditional genetic tests. These tests, which focus on inherited conditions like FVL and prothrombin G20210A mutations, can cost over $500 per individual (35). When scaled to the millions of women considering or already using COCs, this cost becomes prohibitively high. The WHO emphasizes that healthcare systems with constrained budgets would struggle to justify such an expense without clear evidence of significant benefits to the general population (28). The FSRH reiterates this concern, highlighting the resource-intensive nature of widespread thrombophilia screening. It argues that such a strategy would require significant infrastructure and funding, diverting resources from other critical areas of contraceptive and reproductive health care (23). The FSRH and CDC further argue that the absolute risk of VTE in COC users is low, despite a 2- to 6-fold increase in relative risk compared to non-users (9, 10). Nevertheless, while the baseline annual risk of VTE is approximately 2 per 10,000 for healthy women, it rises to 5–16 per 10,000 with COC use (3, 19) leading to an estimate of 22,925 COC-associated VTE events per year in Europe and 14,695 cases in the US.

Another concern raised by guidelines is the limited utility of traditional thrombophilia screening methods. Genetic tests often fail to identify acquired risk factors, such as COC-induced activated protein C (APC) resistance, which further diminishes their value in guiding contraceptive decisions (16, 23, 28–30). The ASH notes that the narrow scope of these tests restricts their ability to meaningfully reduce VTE incidence, particularly when used in isolation (29). The FSRH underscores that even among women with detectable thrombophilia mutations, most will not experience a VTE event during COC use. It is also stated that screening these individuals could lead to unnecessary anxiety and reduced uptake of effective contraception, thereby increasing the risk of unintended pregnancies and their associated complications (23). Nevertheless, Hugon-Rodin et al. (36) investigated the synergistic effect of COC and thrombophilia in a cohort of 2,613 women who experienced their first VTE event (36). To study whether COC use interacts with, e.g. FVL status, a standard measure of synergic index was used corresponding to the ratio between the relative risk of VTE under both exposures, and the product of corresponding relative risks under each one. There was a positive interaction if this index was greater than one. So, assuming e.g. that the risk of VTE is 4-fold increased by FVL and 3.5-fold increased by COC use, a synergy index of 1.0 would mean perfect multiplicative interaction, i.e., relative risk of 4 x 3.5 x 1.0 = 14 in FVL carriers who use COCs. If this relative risk is higher than 14, there is a positive interaction. They observed that the synergistic effect between FVL and COCs varied by progestogen type with COCs containing third generation progestogens or drospirenone or cyproterone acetate being the more at risk with synergy index comprised between 1.63 and 3.13 (36). Khialani et al. (24) further quantified the combined effects of genetic risk factors (e.g., FVL, prothrombin G20210A mutation) and different types of COCs on VTE risk (24). Among women with these mutations, COCs containing levonorgestrel showed the lowest joint risk of VTE, with odds ratio ranging from 7.4 (95% CI: 5.4–10.2) to 24.8 (95% CI: 12.3–50.0) depending on the specific mutation. In comparison, gestodene-containing COCs had odds ratio ranging from 11.7 (95% CI: 7.2–19.1) to 30.9 (95% CI: 10.6–89.9), and desogestrel-containing COCs exhibited odds ratio between 14.6 (95% CI: 9.7–21.9) and 32.6 (95% CI: 13.2–80.6). COCs containing cyproterone acetate showed the highest joint risk, with odds ratio ranging from 15.5 (95% CI: 9.7–24.9) to 44.4 (95% CI: 16.9–116.3) (24). Together, these studies provide compelling evidence that the type of progestogen in COCs and genetic predispositions are key determinants of VTE risk. They support that a test able to encompass both genetic and COC-induced procoagulant status would better reflect the true prothrombotic status than isolated genetic testing. This reinforces the necessity of individualized contraceptive counseling, prioritizing safety in women with known or potential thrombophilia risk factors and those who may overrespond to EE.

**Figure 1:**
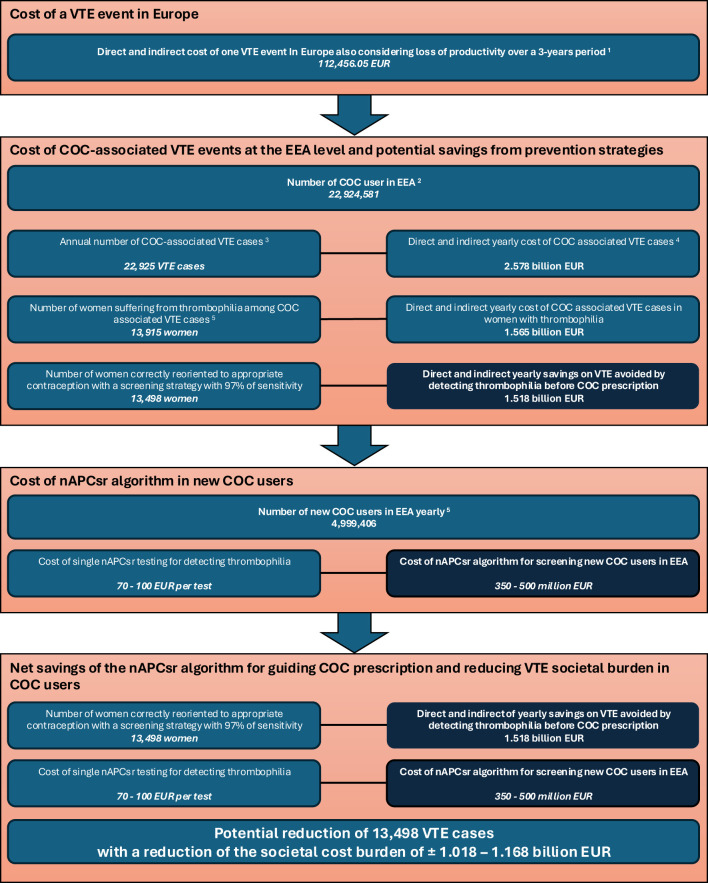
Simulation of the nAPCsr algorithm implementation into clinical practice and the potential annual savings on VTE cases burden cost for prescribing COC in Europe. ^1^Estimated according to Gustafsson et al. (6) and indexed according to the European Harmonised Index of Consumer Prices (HICP) – Health (base 2015: 100 – index 12/2024: 119.8). The cost of a single VTE event is based on Gustafsson data, which calculates the financial impact over a 3-year period. Longer term data are not available but may further increase the cost. ^2^Estimated according to MacDaid et al. (18) and extended to the European Economic Area (EEA). ^3^Based on an annual incidence of 10/10,000 women-year according to McDaid et al. and Khialani et al. (18, 19). ^4^The annual cost takes into account the 3-year management of thrombotic events according to Gustafsson data ^5^Calculated based on data from the START registry reporting that 60.7% of women suffering from COC-associated VTE were thrombophilia positive (22). ^5^Based on Danish data extracted from Khialani et al. (19). COC, combined oral contraceptive; EEA, European Economic Area; nAPCsr, normalized Activated Protein C sensitivity ratio; VTE, venous thromboembolism.

Another point raised by the guidelines is equity which is a key consideration in all guidelines. Universal thrombophilia screening could exacerbate disparities in access to contraception, particularly for women in low-resource settings or those unable to afford the additional costs associated with testing. The WHO and FSRH emphasize the importance of maintaining equitable access to COCs, which are highly effective in preventing unintended pregnancies and improving reproductive health outcomes (23, 28). However, it is crucial to recognize that recent advancements in low-cost, phenotypic screening tools such as the nAPCsr assay offer an opportunity to reconcile equity with effective risk management. The implementation of nAPCsr testing, with a cost significantly lower than traditional genetic screening (around 70 to 100 EUR per test), could alleviate concerns about passing costs onto users while improving clinical outcomes. Economic analyses have demonstrated that targeted screening could lead to a significant reduction in COC-associated VTE cases, translating into substantial healthcare savings that could potentially fund the screening program itself (**Figure 1**). Furthermore, by identifying both inherited and acquired thrombophilia risks, nAPCsr provides a more comprehensive assessment, ensuring that the benefits of safe contraceptive use are accessible to a broader population without disproportionately disadvantaging those in low-resource settings.

The guidelines also point to the low prevalence of thrombophilia mutations in the general population as a reason to discourage universal screening before prescribing COCs. Nevertheless, FVL, the most common inherited thrombophilia, is present in approximately 5–7% of the Caucasian population, while prothrombin mutations occur in 2–3% (37, 38). Combined, these conditions affect approximately 8-9% of the population, which guidelines argue is insufficient to justify routine testing (23, 28–30). However, this argument underestimates the combined and supra-additive burden of inherited and acquired risks, such as APC resistance induced by COCs as depicted in detail above and in recent literature (24, 36, 39–44). The START registry has also shown that over 60% of women with COC-associated VTE have detectable thrombophilia conditions, challenging the notion that these conditions are rare in this context (22).

Despite these longstanding recommendations, advancements in low-cost, phenotypic screening tools like the nAPCsr assay provide an opportunity to revisit these guidelines and current practice. At an estimated cost of 70 EUR per test, the nAPCsr offers a significant reduction in expenses compared to traditional genetic tests while providing broader utility by detecting both inherited and acquired risks (31, 34). This dual functionality enhances its clinical value, particularly for identifying acquired APC resistance, which accounts for a substantial portion of COC-associated VTE cases (45–47). Economic modeling also supports the possibility to reevaluate these guidelines (**Figure 1**). Expanding the results of the START registry (22) and the synergy index of Khialani et al. (24) to the EEA population, the detection of the 60% of women suffering from COC-associated VTE with thrombophilia and the reorientation to appropriate contraceptive methods could save approximately 1.018 to 1.168 billion EUR annually (**Figure 1**). These savings could offset the costs of a comprehensive screening program, making it not only feasible but also cost-effective over time.

Thus, although the WHO, CDC, ASH, and FSRH guidelines discourage routine thrombophilia screening before prescribing COCs based on concerns over cost, low prevalence of thrombophilia mutations, and limited utility of traditional tests, these positions are increasingly challenged by advances in screening technology like the nAPCsr (**Table 1**) and a deeper understanding of the combined burden of inherited and acquired risks. The introduction of affordable and comprehensive screening methods like the nAPCsr assay could transform the cost-benefit equation, enabling more personalized and effective contraceptive care. Revisiting these guidelines to incorporate emerging evidence and innovations is essential to improving the safety and accessibility of contraception for all women.

## The rationale behind developing a screening test for guiding contraception prescription

The interplay between COCs, genetic predispositions, and acquired risk factors emphasizes the need for advanced, targeted screening methods. Emerging tools such as the nAPCsr assay, which can combine the advantages of genetic and metabolic profiling by assessing the pre-existing coagulopathies and phenotypic response to COCs (31), offer a promising pathway for reducing the societal burden of COC-associated VTE. As discussed above, a substantial body of evidence highlights the limitations of traditional risk assessment methods, such as reliance on family history (23), which fails to identify a significant portion of high-risk individuals (22). The nAPCsr assay differs fundamentally from the thrombomodulin (TM)-based ETP ratio developed by Stago (see the review by Tripodi, A. for further details (48)) primarily due to its targeted inhibition level and ability to discriminate between different COCs. The TM-based ETP ratio assay is designed to achieve 50% inhibition of thrombin generation in a reference population, which results in a wider variation in normal individuals and a limited dynamic range, i.e. from 50% to 0% inhibition. This reduces its sensitivity to detect subtle differences in APC resistance induced by different contraceptive formulations. In contrast, the nAPCsr method targets 90% inhibition (31), which significantly enhances test sensitivity and the ability to differentiate between COCs, making it a more robust tool for population-level assessment of thrombotic risk. Given these advantages, the transfer of the nAPCsr assay to the ST-Genesia platform, an automated thrombin generation analyser, was crucial as it allows for full automation, improved reproducibility, and accessibility in routine clinical laboratories, thereby facilitating its use in regulatory and clinical decision-making (49). Manufacturers are working on the development of a nAPCsr CE-marked kit to implement on the ST-Genesia system (50).

The choice of COC formulation also influences VTE risk, with those containing EE combined with less androgenic progestins, such as desogestrel, gestodene, or drospirenone, exhibiting a higher thrombotic risk than second-generation formulations with levonorgestrel (10). This disparity arises from the differential modulation of EE’s procoagulant effects, with less androgenic progestins failing to counteract EE-induced hepatic synthesis of clotting factors (39). Furthermore, a recent meta-analysis also highlighted that natural estrogens may reduce the risk of VTE by ±50% when compared to EE-levonorgestrel, results which were confirmed in a pharmacovigilance database (32, 33) stressing the point that the problem is not related to the progestin but to EE, especially when administered to inappropriate populations. These findings emphasize the urgent need for personalized contraceptive strategies informed by an individual’s genetic, metabolic, and thrombotic risk profile.

By integrating nAPCsr screening into contraceptive counseling, healthcare providers can objectively assess an individual’s risk and guide contraceptive decisions accordingly. Women identified as high-risk, either due to known thrombophilia or elevated nAPCsr due to excessive response to EE (**Table 2**), can be redirected towards safer alternatives, such as POPs or COCs formulated with natural estrogens like estradiol (E2) or estetrol (E4) (32, 33).

**Table 2:** Common genetic mutations correlated with COC-associated VTE and performance of the nAPCsr for detecting these conditions.

Genetic mutation	Prevalence in the population	Relative risk for VTE in absence of COC	Implication in COC-associated VTE	Detectable by the nAPCsr?
Classical thrombophilia screening (38)				
Factor V Leiden *rs6025* *Allele A*	Heterozygous: 5-7% Homozygous: 0.1%	Heterozygous: 7 Homozygous: 80	The FVL causes factor V resistance to the anticoagulant action of APC. It increases the risk of COC-associated VTE in a synergistic manner (24, 36). Note: Other less frequent mutations on the F5 gene have also been identied and are FV Cambridge, FV Hong Kong, FV Bonn, FV Nara, FV Besançon, and FV Liverpool (34).	Yes — the nAPCsr can detect resistance towards APC which is typically expressed with FV mutations and its supra-additive effect with COC (31).
Prothrombin G20210A *rs1799963* *Allele A*	Heterozygous: 2% Homozygous:0.02%	Heterozygous: 3-4 Homozygous: 30	The G20210A mutation increases the level of factor II (prothrombin), thereby increasing the procoagulant status. The risk of COC-associated VTE is increased in a synergistic manner (24, 36).	Yes — the nAPCsr being derived from a thrombin generation test, the test is able to detect the excess in prothrombin and the resistance towards APC associated with COC (61).
Protein C deficiency *rs9574* *Allele G*	Heterozygous: 0.2 – 0.4% Homozygous: 1 in 500,000 to 1 in 750,000 live births	Heterozygous: 15 Homozygous: not estimated - events occur within hours post-delivery in neonates	Protein C is a key component of the natural anticoagulant pathway that downregulates thrombin generation by inactivating coagulation factors Va and VIIIa. A deficiency in protein C results in a prothrombotic state, which can be exacerbated by COCs due to their procoagulant effects (62)	Not completely — The nAPCsr, by adding external APC into the testing system is not highly sensitive to endogenous protein C variations. However, severe form of protein C deficiency are detected in neonates while mild form of protein C deficiency are present in less than 5% of patients with COC-induced VTE (63).
Protein S deficiency *Multiple mutations involved*	Heterozygous: 0.03 – 0.1% Homozygous: 1 in 500,000	Heterozygous: 10 Homozygous: not estimated-life-threatening complications in infancy	Protein S is a key component of the natural anticoagulant pathway and forms a complex with activated protein C that downregulate thrombin generation by inactivating coagulation factors Va and VIIIa. A deficiency in protein S results in a prothrombotic state, which can be exacerbated by COCs due to their procoagulant effects. Both lead to APC resistance (34).	Yes — The nAPCsr is able to detect both protein S deficiency and COC-associated prothrombotic effects. Both are leading to APC resistance that can be detected by the nAPCsr (31).
Antithrombin deficiency *Multiple mutations involved*	Heterozygous: 0.02 – 0.2 Homozygous: Approximately 1 in 500,000 to 1 in 750,000 live births	Heterozygous: 50 Homozygous: not estimated	Antithrombin is a key endogenous inhibitor of thrombin and factor Xa, playing a critical role in maintaining hemostatic balance by limiting clot formation. Deficiency of antithrombin—whether hereditary or acquired—leads to a pronounced prothrombotic state due to insufficient inhibition of thrombin generation. COCs exacerbate this hypercoagulable environment by further reducing AT activity and increasing levels of procoagulant factors such as factor VIII and fibrinogen, while simultaneously decreasing natural anticoagulants like protein S (64).	Yes — the nAPCsr being derived from a thrombin generation test, the test is able to detect the lack of thrombin inhibition by antithrombin (64).
Uncertain thrombophilia screening (65) and genetic variants associated with inappropriate estrogenic response (17)				
Fibrinogen Gamma (FGG) rs2066865 Allele T	26-30%	1.6	The rs2066865 mutation has been associated with the formation of denser, less porous fibrin clots that are more resistant to fibrinolysis (66). COCs further promote the formation of such clots by increasing prothrombotic factors, including factor VIII and fibrinogen. The presence of the mutation worsens clot stability, making it more difficult for the body to break down clots, thus increasing thrombotic risk.	Yes — the nAPCsr captures the effect of the rs2066865 mutation by quantifying the plasma response to activated protein C (APC). In carriers of the FGG H2 haplotype (tagged by rs2066865), reduced fibrinogen γ’ levels lead to impaired APC sensitivity, reflected by a higher residual thrombin generation. COCs exacerbate this state by elevating procoagulant factors, further increasing APC resistance and creating a synergistic risk for thrombosis (67).
Non-O blood group *rs8176719* *Allele G* *rs8176750* *Allele C*	55-57%	2.0	Non-O blood group have higher level of FVIII which, in combination with the procoagulant impact of COC can further increase the risk of VTE.	Yes — the nAPCsr being derived from a thrombin generation test, the test is able to detect the excess in FVIII and its associated increased in thrombin generation and the resistance towards APC associated with COC (68).
F11 *rs2289252* *Allele T*	30-40%	1.2	The rs2289252 polymorphism is a single nucleotide variant located in the F11 gene, which encodes Factor XI, a crucial component of the intrinsic coagulation pathway. A study analyzing women using COCs reported that the presence of the rs2289252-A allele (equivalent to the C allele in some studies due to strand orientation) was associated with an increased risk of VTE, with an odds ratio (OR) of 1.6. Furthermore, the combination of this allele with non-O blood groups elevated the risk to an OR of 4 (69, 70).	Plausible — rs2289252 might indirectly influence the results of thrombin generation tests or the nAPCsr due to its effect on Factor XI levels.
KNG1 *rs710446* *Allele C*	41%	1.2	KNG1 encodes high molecular weight kininogen (HK), which plays a critical role in the contact activation pathway of coagulation by acting as a cofactor for the activation of prekallikrein and factor XII (FXII). This pathway is involved in the initiation of thrombin generation and clot formation (71). Estrogen in COCs increases the levels of coagulation factors (such as factors VII, VIII, and X) and decreases the levels of anticoagulant proteins (such as protein S). In women carrying the C allele, the combined effect of higher FXI levels due to rs710446 and the procoagulant changes induced by estrogen can synergistically elevate thrombotic risk (18, 72).	Plausible — While the rs710446 mutation is not directly mentioned in the context of thrombin generation tests, it is plausible that this mutation could influence thrombin generation results and therefore nAPCsr, particularly at low tissue factor concentrations, due to its association with factor XI levels.
SLC44A2 *rs2288904* *Allele G*	79%	1.2	The rs2288904 polymorphism is a single nucleotide variant in the SLC44A2 gene, which encodes the choline transporter-like protein 2 (CTL2). It defines the human neutrophil antigen (HNA) system HNA-3 (73).	Not known
TSPAN15 *rs78707713* *Allele T*	88%	1.3	The exact mechanism remains unclear, the association of rs78707713 with VTE risk suggests that TSPAN15 may play an unexpected role in thrombosis pathophysiology, potentially involving novel biological pathways distinct from traditional coagulation factors. Further research is needed to elucidate the specific molecular mechanisms by which this genetic variant increases thrombosis risk (73).	Not known
CYP3A4 *Haplotype A & B*	± 15%	No link with VTE outside COC	The CYP3A4 enzyme is involved in the metabolism of EE. The rs2242480 and rs6945984 alleles could lead to decreased metabolism of EE, potentially increasing its plasma levels and overall estrogenic effect (74, 75)	Yes — the nAPCsr is sensitive to the level of estrogens and their impact on the liver, the level of coagulation factors and the associated APC resistance (60).
CYP2C9 *rs1799853* *Allele T*	13%	No link with VTE outside COC	The CYP2C9 enzyme is involved in the metabolism of EE. The rs1799853 (T) allele could lead to decreased metabolism of EE, potentially increasing its plasma levels and overall estrogenic effect (18, 76).	Yes — the nAPCsr is sensitive to the level of estrogens and their impact on the liver, the level of coagulation factors and the associated APC resistance (60).
UGT2B7 Haplotype D	Not known	No link with VTE outside COC	Haplotype D refers to a specific combination of genetic variants (alleles) in the UGT2B7 gene, which encodes the UDP-glucuronosyltransferase 2B7 enzyme. This enzyme is involved in the metabolism and glucuronidation of various endogenous hormones, drugs, and xenobiotics including EE (51).	Yes — the nAPCsr is sensitive to the level of estrogens and their impact on the liver, the level of coagulation factors and the associated APC resistance (60).
*SUGCT* *rs4379368* *Allele T*	53%	Not estimated	The exact mechanism is not explicitly stated, rs4379368 is located near the C7orf10 gene (also known as SUGCT) which encodes for succinic HMG coenzyme A transferase and is associated with glutaric acid metabolism. This genetic variant might influence metabolic pathways that could affect coagulation or other factors relevant to VTE risk (77).	Not known

APC, activated protein C; COC, combined oral contraceptive; E2, estradiol; E4, estetrol; EE, ethinylestradiol; FVL, Factor V Leiden; nAPCsr, normalized activated protein C sensitivity ratio; POC, progestin only contraceptive; VTE, venous thromboembolism.

Given that more than 60% of COC-associated VTE cases occur in women with positive thrombophilia screening, targeted interventions could significantly reduce VTE incidence. Identifying and mitigating risks in 97% of these cases could lower the overall burden by more than 55%, reducing the absolute burden of VTE by 13,500 cases at the EEA level (22) (**Figure 1**). Additionally, the nAPCsr assay’s ability to detect acquired APC resistance offers further opportunities to address the residual 40% of cases potentially linked to EE over-responsiveness (31). Indeed, the role of genetic variations in EE metabolism underscores the need for personalized approaches. Variants in the CYP3A4 gene, which governs the first-pass hepatic metabolism of EE, significantly modulate the bioavailability and procoagulant activity of COCs. Carriers of pro-thrombotic CYP3A4 haplotypes, such as haplotype B, face a markedly elevated VTE risk (OR:1.86, 95%CI:1.17–2.94) when using EE-containing formulations (51). Incorporating phenotypic CYP3A4 capacity to metabolize EE into screening protocols could refine risk stratification and guide the selection of safer contraceptive options at the individual level.

Advancing beyond traditional family history-based risk assessments, the integration of nAPCsr screening or cost-effective genetic testing, and tailored counseling into contraceptive care offers a transformative opportunity to reduce the societal and economic burden of COC-associated VTE. By adopting a comprehensive, decision share-making, patient-centred care, healthcare systems can enhance patient safety, optimize resource allocation, and mitigate the profound impact of preventable thrombotic events.

## The potential medical and societal benefits of a low-cost thrombophilia screening test before prescribing combined oral contraceptives

**Figure 2:**
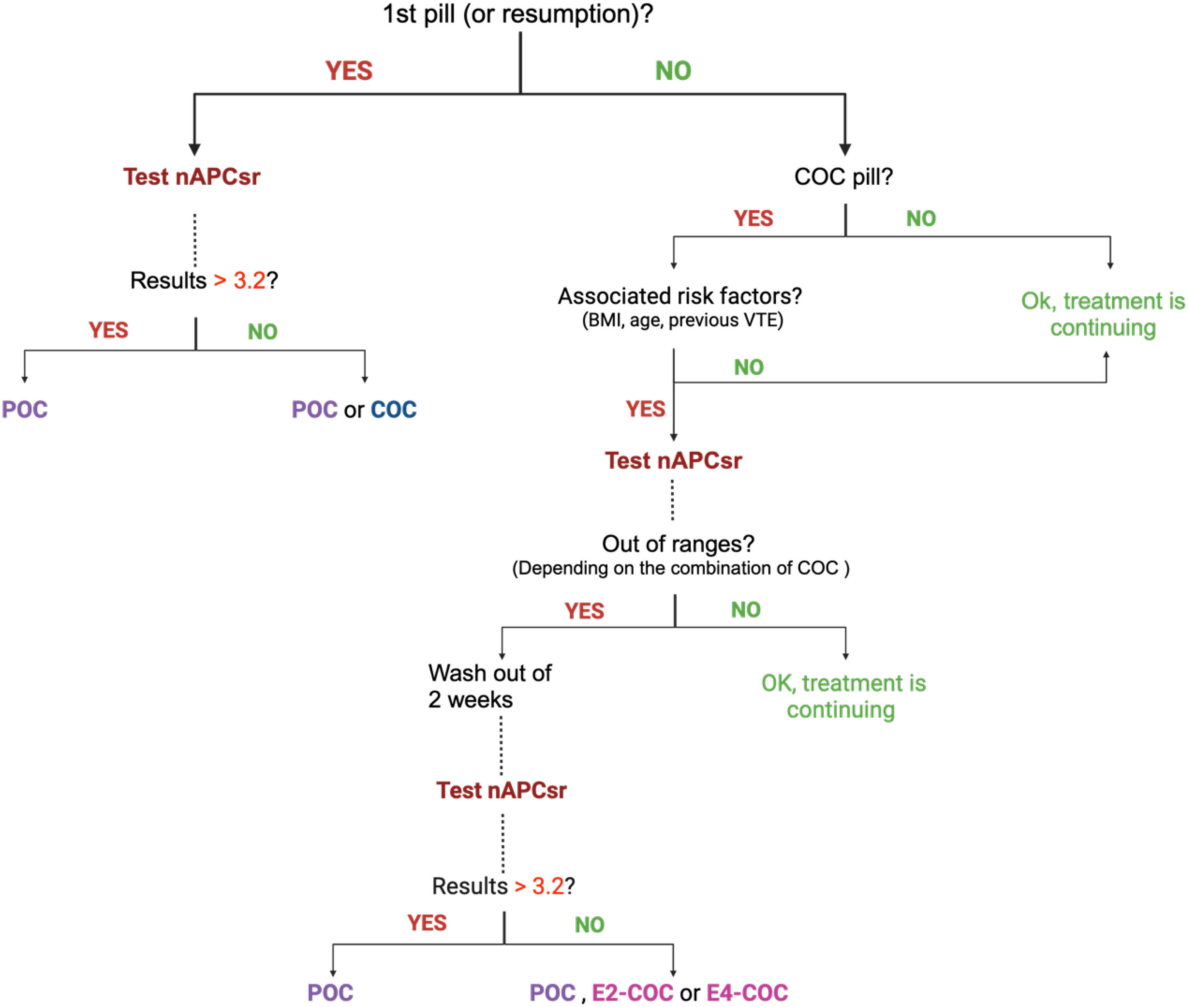
Normalized APC sensitivity ratio algorithm for prescribing combined oral contraceptives in first time users or switchers and in current users. BMI, body mass index; COC, combined oral contraceptive; E2, estradiol; E4, estetrol; nAPCsr, normalized activated protein C sensitivity ratio; POC, progestin only contraceptive.

The implementation of a comprehensive thrombophilia screening strategy, like the nAPCsr assay, represents a transformative approach to mitigating the burden of COC-associated VTE. This strategy is designed to address both new and existing COC users in Europe, ensuring comprehensive risk stratification and informed contraceptive choices. The proposed model includes two tests for new users, i.e. one before initiating COCs to rule out inherited thrombophilia and another after 1–2 cycles to identify acquired resistance to APC due to over-responsiveness to the estrogenic component (**Figure 2**). Additionally, screening women already on COCs, who comprise approximately 30% of the European population aged 14–49, could extend the benefits of this preventive approach.

The economic implications of this strategy are significant yet justified. Considering Danish data (19), approximately 5 million new COC users per year in the EEA would require to be screened representing a cost of 350–500 million EUR. Among them and based on an estimated thrombophilia prevalence of 8-9% in Europe (37), 399,952 women will be identified as not eligible to COC and will be reoriented to appropriate contraceptive methods. Therefore, for the residual 4,600,000 women, a second nAPCsr testing could be required 1 month after treatment initiation to ensure they are not high EE-responders. This translates into an additional cost of 322–460 million EUR to identify high responders to the estrogenic component of COC. Further investigations are required to clearly identify the benefit of this second screening but only considering the CYP3A4 haplotype B, which has a prevalence of 13% in the European and 77% in the African ancestries (52) and is associated with an increased risk of VTE compared to users of COC without CYP3A4 mutation (OR:1.86, 95%CI:1.17–2.94) (51), an additional 568,800 women in the EEA could be reoriented to more appropriate contraceptive solutions (considering that 2% of the EEA population is from African origin). The reduction in the risk of VTE among these 568,800 women from ±20/10.000 women-year to ±5/10,000 women-year if they are switched to natural estrogens (32, 33) may further reduce the number of VTE by ±850 cases, representing 96 millions of EUR of economy of the VTE burden.

Considering that the nAPCsr is able to detect with 97% sensibility patients with FVL or with prothrombin mutation, the two more prevalent thrombophilia in the European population (53), we can estimate that near 59% of the population suffering from COC-associated VTE will be correctly oriented to less thrombotic contraceptive solutions. This can lead to an estimated prevention of 13,498 VTE cases annually (**Figure 1**). Given the average cost of managing a single VTE event over a 3-years period at 112,456 EUR (**Figure 1**), this reduction translates into 1.518 billion EUR in annual savings across the EEA healthcare system. Over a decade, the cumulative savings would reach more than 10 billion EUR, more than offsetting the initial investment in screening and providing a sustainable financial benefit.

These economic reflections challenge the current stance of organizations, which advises against routine thrombophilia screening before initiating COCs due to perceived high costs and low prevalence of thrombogenic mutations or alteration in the metabolism of EE. The traditional approach of comparing the one-time cost of genetic testing against benefits calculated per person-year inherently underestimates the long-term value of screening. As highlighted by Vernon et al., this methodology neglects the extended duration of COC use, often spanning several years, and fails to account for the lifetime benefits of identifying thrombophilia conditions (54). When considered alongside the nAPCsr assay’s ability to detect not only inherited thrombophilia but also acquired APC resistance, the utility of screening becomes undeniable on a societal point of view.

Importantly, this strategy aligns with the principles of personalized medicine, providing tailored contraceptive recommendations based on individual risk profiles (35). Women identified as high-risk can be redirected to safer alternatives, such as progestin-only pills, intrauterine devices or natural estrogen-based COCs, minimizing their thrombotic risk while preserving contraceptive efficacy. Importantly, although international guidelines recommend discontinuing COC immediately or after cessation of anticoagulant therapy when treated for a VTE with the aim to prevent recurrences, a sizable proportion of women either continues or starts COC use after a first VTE (55, 56). Evaluation of the nAPCsr in these women could also be of interest to decipher the root cause of the prothrombotic profile. Screening also empowers women by providing critical health information that extends beyond contraception, allowing them to make informed decisions during other high-risk life stages, such as pregnancy or surgery. The inclusion of nAPCsr testing in this model enhances its practicality and affordability, addressing both the economic barriers to screening and the limitations of traditional genetic tests, which can exceed 500 EUR per individual.

## Limitations

While this preliminary economic assessment offers a relevant and potentially high-impact strategy for reducing the burden of COC-associated VTE, further refinements are warranted. Although a detailed cost-effectiveness analysis remains necessary to comprehensively evaluate the financial feasibility of the proposed approach, the current economic model provides robust and meaningful initial estimates, supported by real-world data on VTE management costs. These preliminary findings offer a strong foundation for future research and policy discussions aimed at improving public health outcomes. Second, while the generalizability of findings may be limited by the predominantly European focus of the data, this region represents a significant portion of the global COC-using population, and its healthcare infrastructure offers valuable insights. Expanding this research to other regions, particularly low- and middle-income countries, would further validate the applicability of the proposed strategy across diverse healthcare settings. Nevertheless, the approach outlined in this manuscript serves as a scalable model adaptable to different healthcare environments.

However, one of the challenges associated with nAPCsr testing is that TGA, including the ETP-based APC resistance assay, require specialized equipment such as a fluorimeter and trained personnel, which may limit immediate accessibility in non-specialized laboratories. Historically, TGA has been predominantly used in research settings rather than for individual patient screening. The need for expert technicians or biologists to ensure proper assay performance has been highlighted as a potential barrier to its large-scale implementation (57). Nevertheless, the recent transition of nAPCsr testing to the ST-Genesia platform represents a major step toward simplifying and automating the process, thereby reducing technical complexity and improving reproducibility (58). Unlike earlier thrombin generation assays, the ST-Genesia offers a standardized, fully automated system that minimizes operator-dependent variability and simplifies routine testing (58). Additionally, the interpretation of nAPCsr results is straightforward, with values expressed on a normalized scale from 0 to 10, making it easier for clinicians to integrate into routine contraceptive risk assessments without requiring extensive coagulation expertise (59).

Furthermore, while nAPCsr provides a functional assessment of thrombin generation and APC resistance, it does not account for other well-established risk factors for VTE, such as body mass index, age, or smoking status. Therefore, clinical decision-making should integrate nAPCsr results with a comprehensive evaluation of patient-specific risk factors to ensure an optimal risk assessment.

Targeted policies that subsidize testing for at-risk populations can further ensure equitable access and prevent disparities in contraceptive safety. Finally, although the manuscript primarily focuses on reducing VTE incidence and healthcare costs, the broader impact on reproductive health outcomes, such as contraceptive adherence and unintended pregnancies, merits further investigation. Additionally, while the potential psychological impact of screening is an important consideration, it can be minimized through proper patient education and counseling, which are integral components of any screening program. By addressing these areas in future studies, the full societal and clinical benefits of thrombophilia screening can be more comprehensively realized.

## Conclusions

In conclusion, the proposed screening strategy represents a potentially cost-effective and impactful intervention for mitigating the burden of COC-associated VTE. Incorporating low-cost and accessible testing into routine contraceptive counseling has the potential to enhance health outcomes for millions of women while generating substantial economic savings. Specifically, annual healthcare savings exceeding one billion EUR across the EEA, with cumulative savings surpassing 10 billion EUR over a decade, underscore the favorable cost-benefit ratio of this initiative. These findings provide strong justification for policy revisions and the incorporation of thrombophilia screening into clinical practice. While the primary focus of this approach is on contraceptive safety, the underlying technology could also hold broader clinical relevance, such as in assessing VTE risk during menopausal replacement therapy, pregnancy and the postpartum period. Future research could explore these additional applications, further reinforcing the cost-effectiveness and clinical utility of this test beyond COC use.

Although further research is warranted to validate the long-term efficacy and economic viability of this approach, current guidelines appear misaligned with emerging scientific evidence and technological advancements. Consequently, regulatory bodies and national healthcare reimbursement systems should prioritize the integration of targeted screening strategies to reduce both the clinical and financial burdens associated with COC-related VTE.

## Data Availability

The original contributions presented in the study are included in the article/supplementary material. Further inquiries can be directed to the corresponding author.
